# Doing *Duo* – a case study of entrainment in William Forsythe’s choreography “*Duo*”

**DOI:** 10.3389/fnhum.2014.00812

**Published:** 2014-10-21

**Authors:** Elizabeth Waterhouse, Riley Watts, Bettina E. Bläsing

**Affiliations:** ^1^The Forsythe CompanyFrankfurt, Germany; ^2^Faculty of Psychology and Sport Science, Neurocognition and Action – Research Group, Bielefeld UniversityBielefeld, Germany; ^3^Center of Excellence – Cognitive Interaction Technology, Bielefeld UniversityBielefeld, Germany

**Keywords:** entrainment, contemporary dance, choreography, multimodal communication, coordination, synchronization, joint action

## Abstract

Entrainment theory focuses on processes in which interacting (i.e., coupled) rhythmic systems stabilize, producing synchronization in the ideal sense, and forms of phase related rhythmic coordination in complex cases. In human action, entrainment involves spatiotemporal and social aspects, characterizing the meaningful activities of music, dance, and communication. How can the phenomenon of human entrainment be meaningfully studied in complex situations such as dance? We present an in-progress case study of entrainment in William Forsythe’s choreography *Duo,* a duet in which coordinated rhythmic activity is achieved without an external musical beat and without touch-based interaction. Using concepts of entrainment from different disciplines as well as insight from *Duo* performer Riley Watts, we question definitions of entrainment in the context of dance. The functions of chorusing, turn-taking, complementary action, cues, and alignments are discussed and linked to supporting annotated video material. While *Duo* challenges the definition of entrainment in dance as coordinated response to an external musical or rhythmic signal, it supports the definition of entrainment as coordinated interplay of motion and sound production by active agents (i.e., dancers) in the field. Agreeing that human entrainment should be studied on multiple levels, we suggest that entrainment between the dancers in *Duo* is elastic in time and propose how to test this hypothesis empirically. We do not claim that our proposed model of elasticity is applicable to all forms of human entrainment nor to all examples of entrainment in dance. Rather, we suggest studying higher order phase correction (the stabilizing tendency of entrainment) as a potential aspect to be incorporated into other models.

## INTRODUCTION

What is entrainment? And how can entrainment in dance be theorized and scientifically measured? Human entrainment has been studied in a variety of contexts including music, dance, motor action, and communication. To promote exchange and evaluate the coherence of human entrainment across research domains, cross-disciplinary language, and a body-specific framework are justified. As a first step, entrainment theory could be framed provisionally as the study of synchronization. Who or what synchronizes? How much? How? Where? And in what case? These are all warranted concerns that will be discussed accordingly in the body of the article.

This article supports the perspective that scientific studies of entrainment can benefit from interdisciplinary collaboration, connecting researchers in the sciences to artists in the professional field. We advocate that a productive method for this is long-term investment in analyzing case studies curated by artists. We report here upon activities initiated in The Forsythe Company’s research platform *Dance Engaging Science*, considering the choreography of *Duo*, one of many renowned works choreographed by William Forsythe (artistic director of Ballet Frankfurt/The Forsythe Company from 1984 to 2015). We recommend that readers familiarize themselves with the dance before reading the article, by viewing the media provided online at http://www.dancelikething.org/Duo.html (video password: Frontiers).

**FIGURE 1:**
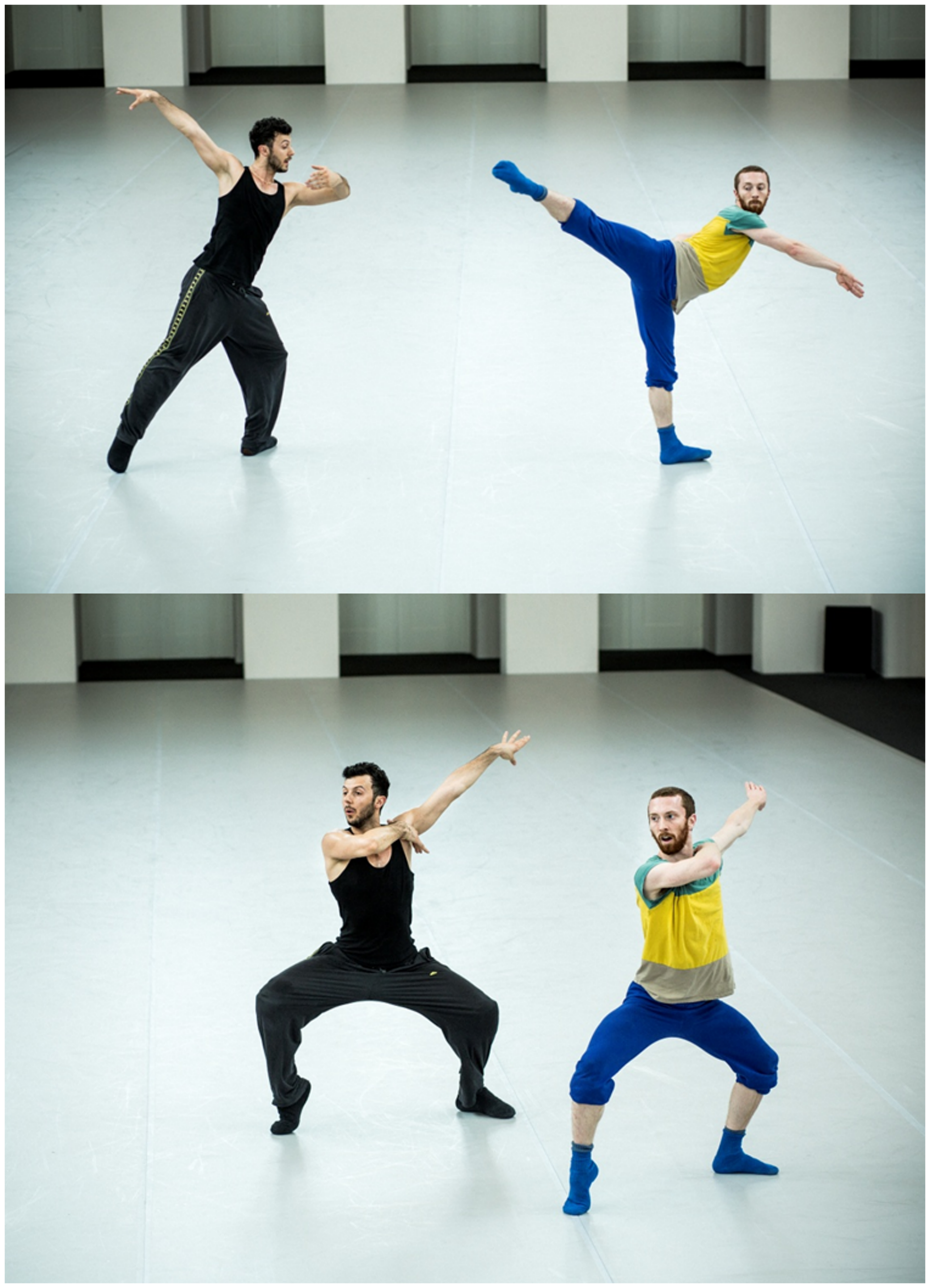
**Brigel Gjoka (left) and Riley Watts (right) during a rehearsal of *Duo* in Dresden in 2012.** Photographs by Dominik Mentzos.

The choreography of *Duo* is aesthetically and scientifically significant. Part of this is the impressive skill of the performers: two vocalizing dancers dancing synchronously and asynchronously without a musical pulse (see **Figure 1**). From visual observation, it appears that this synchronization is seemingly accurate, musical, and even conversational. As Schachner (2013) demanded of her own observations of birds moving “seemingly in time with the musical beat,” we also ask what instruments of measurement and data analysis can reveal about the process of synchronizing in *Duo*. Working with *Duo* as a case study, our motivation was to develop interdisciplinary approaches to meaningful measurement and theoretical understanding of the phenomenon of entrainment between dancers.

In the following sections, we introduce the language for entrainment from different perspectives (see section “Understanding Entrainment”) to ground our case study in relevant state-of-the-art knowledge. In section “*Duo* – A Case Study of Entrainment,” we present our case study, focusing extensively on the perspective of performer Riley Watts. We then consider the learning and rehearsal process that we regard as a noteworthy precondition for successful entrainment between the performers in section “Entrainment as Learned, Rehearsed Practice.” In section “Meaningful Measurement of Entrainment in *Duo*,” we suggest that entrainment in dance should be studied in multiple layers. Finally, we propose how our hypotheses regarding entrainment in *Duo* could be tested empirically and propose a model of elasticity.

The ability to coordinate rhythmic movement in *Duo* can be viewed as a multimodal and ecological process of shared intentionality, accompanied by emotional bonding, and learned through repeatedly rehearsing and performing choreography. Entrainment in *Duo* requires the integration of multimodal sensory cues with multiple cognitive processes that enable the dancers to mutually entrain to each other, thereby showing typical characteristics that have been ascribed to entrainment in communication rather than in music or dance. Expanding the general definition of entrainment as coordinated rhythmic activity (Phillips-Silver et al., 2010), we specify entrainment in *Duo* as the coordinated interplay of motion and sound production by active agents (i.e., dancers) in the field.

## UNDERSTANDING ENTRAINMENT

### SCIENTIFIC APPROACHES TO ENTRAINMENT

Entrainment has been studied in mechanical, animal, and human systems (see, e.g., Repp and Su, 2013). Clayton (2012) marks this, emphasizing that entrainment is not a single phenomenon or behavior, but rather “an abstraction that can be used to make sense of many different phenomena.” The origin of entrainment studies is the mechanical consideration of how two or more coupled rhythmic oscillators interact, adjusting over time to align in period (Clayton et al., 2005). The work of the Dutch physicist Christiaan Huygens is cited as the first to refer to entrainment. Huygens posited that the “odd kind of sympathy” between pendulums of double clocks might be caused by an “imperceptible movement” of the beam between them, enabling out-of phase synchronization to develop (see Bennett et al., 2002).

Phillips-Silver and Keller (2012) define human entrainment as “the spatiotemporal coordination between two or more individuals, often in response to a rhythmic signal.” To enable cross comparison of human entrainment across domains and in situations without a rhythmic signal, Phillips-Silver et al. (2010) previously proposed a unified framework of entrainment aspects, framing human entrainment as the *coordination of rhythmic movement*. For the purpose of developing an interdisciplinary relevant definition of entrainment for the context of contemporary dance, we take this operational definition of entrainment as our starting point.

Phillips-Silver et al. (2010) observe that entrainment is predicated on the abilities to perceive and produce rhythmic action and real-time integration between sensory and motor systems. The authors highlight that entrainment often occurs in more complicated contexts than with an isochronous pulse, to a wide range of tempi, and that it involves sensorimotor activity across multiple sensory modalities. Clayton (2012) and similarly Phillips-Silver et al. (2010) propose different levels of entrainment: self-entrainment (intra-individual) within a particular human being as the coordination of body parts, mutual entrainment (inter-individual) between two persons, and social entrainment within (intra-group) or even between (inter-group) groups. Clayton (2012) notes that while these levels may build upon each other, self-entrainment might not necessarily be fundamental. Phillips-Silver and Keller (2012) suggest that the roots of entrainment might be developed in infancy via mimesis with a caretaker, and propose temporal and affective components of entrainment, the latter involving interpersonal bonds as well as the pleasure in moving in time with others.

In order to take a closer look at the basic definition of entrainment as coordination of rhythmic movement, the underlying concepts of rhythm and coordination have to be regarded more closely. The term *rhythm* is used in this context for signals with differing periodicity or predictability, “from musical rhythm to conversation and language processing, non-verbal communication, gesture, play and sharing of attentional gaze” (Phillips-Silver et al., 2010). It has to be noted that the term rhythm is not defined consistently throughout the literature, rather, there are different relevant working definitions of rhythm used in different disciplines, including physics, music, and cognitive psychology. Clayton (2012) describes rhythmical systems as showing periodic or quasi-periodic oscillatory activity, being self-sustaining (i.e., independent of external rhythms), and, in order to interact, to be coupled. Large (2000, 2010) proposed a neurodynamic model of coupled oscillators that synchronize with external rhythmic patterns, demonstrating entrainment, synchronization and temporal expectancy based on neural resonance.

For disciplines generally related to human action (such as psychology), definitions of rhythm typically refer to the processing and effects of rhythmic signals. Cummins (2009) proposes a strongly enactivist definition of rhythm as “an affordance for the entrainment of movement,” stating that “there can be no effective rhythm in the absence of the potential for physical movement.” In the context of music, rhythmic behavior refers to the “ability to process and respond to a regular pulse” (Phillips-Silver et al., 2010). Large and Snyder (2009) define musical rhythms as “complex, temporally structured sequences of acoustic events” and state that even though “rhythms of music are not periodic,” “in most musical rhythms people perceive periodicity, called *pulse* or *beat*.” Bispham (2006) describes musical rhythmic behavior as based on a set of subskills including timing abilities, periodic and non-periodic movement, pulse perception, action-perception coupling, and error correction mechanisms. These perspectives contrast the concept of rhythm to that of pulse, which has been defined as “endogenous periodicity, a series of regular recurring, precisely equivalent psychological events that arise in response to a musical rhythm” (Large and Snyder, 2009), or “subjectively experienced isochrony” (Su and Pöppel, 2012). Merker et al. (2009) identify this isochrony as the basic principle of human entrainment to auditory stimuli.

Phillips-Silver and Keller (2012) refer to *coordination* as when “movements between co-actors might be coupled in a synchronized or complementary fashion.” Coordination is thus viewed as a basic principle underlying human interaction in social contexts. Knoblich et al. (2011) define entrainment as a specific source of emergent coordination, one key factor in joint action. *Joint action*, according to Sebanz et al. (2006), is defined as “any form of social interaction whereby two or more individuals coordinate their actions in space and time to bring about a change in the environment.” In their comprehensive work on joint action, Knoblich et al. (2011) differentiate between social situations in which people coordinate their movement unwittingly despite their intention to ignore each other (emergent coordination), situations in which people coordinate as part of joint action but without specific advantage of doing so (emergent coordination in joint action) and situations in which coordination is deliberate and serves a purpose for achieving the goal of the joint action (planned coordination). In joint action, emergent coordination based on inter-individual entrainment of (often unintentional) movements has been proposed to serve cognitive or social functions that facilitate cooperation (Knoblich et al., 2011). Well-studied examples are entrainment of body sway during conversation (e.g., Shockley et al., 2003, 2007; Fowler et al., 2008) or entrainment of stepping patterns while walking together (e.g., Van Ulzen et al., 2008; Miles et al., 2009).

Emergent coordination based on unintentional inter-individual entrainment has been studied in people swinging a pendulum (Schmidt et al., 2012), tapping with their fingers (e.g., Oullier et al., 2008; Konvalinka et al., 2010; see Repp, 2005; Repp and Su, 2013 for review), swinging a leg (Schmidt et al., 1990) and walking (Van Ulzen et al., 2008; Repp and Su, 2013). The underlying processes have been explained by applying dynamical systems theory (e.g., Schmidt and Richardson, 2008), interpreting the coordinated action in terms of coupled oscillators (“clocks hanging on the same wall,” see Bennett et al., 2002). Several studies have investigated emergent coordination of rocking frequencies in two or more people sitting in rocking chairs (Richardson et al., 2007; Demos et al., 2010, 2012; Valdesolo et al., 2010). Emergent coordination based on group entrainment has been studied by Neda et al. (2000) who showed that members of theater audiences tend to alternate between slowing down their individual clapping frequencies to clap in unison and de-synchronizing again to increase over-all clapping loudness (thereby showing characteristics of a dynamical system with two attractors).

Planned coordination has been studied extensively in music (e.g., Repp and Keller, 2004; Keller, 2008; Goebl and Palmer, 2009) and (less extensively) in dance (see Sevdalis and Keller, 2011; Bläsing et al., 2012). Knoblich and Sebanz (2008) point out that specifically in music, art, and sport, joint action depends on inter-individual entrainment, which is based on and interacts with shared intentionality (specifically with the intention to synchronize). This is well illustrated by a remarkable study on coordination and synchronization within a flamenco ensemble consisting of dancers and musicians (Maduell and Wing, 2007). Based on studies of entrainment in dance (Naveda and Leman, 2010; Maes et al., 2012; Van Dyck et al., 2013), Leman (2012) asserts that entrainment is an embodied and ecological phenomenon, complexified by interaction of sensorimotor control, and action-perception loops. Naveda and Leman (2010) studied Charleston and Samba dancers, looking at spatiotemporal signatures of gross-motor and fine-motor gestures to understand the extent to which gesture may influence entrainment between dancers. Leman (2012) corroborates that entrainment may depend on gestures, proposing to study entrainment not purely temporally, but rather in an embodied context, including “a spatiotemporal dimension that is rooted in bodily gestures.” Emphasizing spatiotemporal aspects of entrainment, Leman (2012) argues that entrainment occurs at different levels and in different forms, naming the levels of context, gesture repertoire, and sensorimotor control. Like Clayton (2012), Leman (2012) recognized objective and subjective aspects of entrainment, relating to objective spatiotemporal measurements vs. subjective accounts of entrainment experiences, intentions, and the role of gestures and corporeal articulations. The author suggests that, in the context of music, it is the practice or rehearsal that aims “at getting synchronization right,” and points out that “in order to musically entrain spontaneously, one has to practice hard.” Geeves et al. (2014) add that rehearsed entrainment between musicians may also serve the purpose of making ensemble performance more robust against distractions, thereby giving individual ensemble members (or soloists) more freedom to improvise. Demos et al. (2014) proposed a dynamical systems approach for studying body movements of musicians while performing, claiming that these relate strongly to the performers’ expressive intentions and have thereby communicative character.

Following the view of Demos et al. (2014) and other authors, it seems crucial at this point to take a closer look at concepts of entrainment in linguistic communication. Phillips-Silver et al. (2010) characterize entrainment in communication as non-pulse-based, in contrast to pulse-based entrainment in music or dance. Entrainment in communication can be observed on different linguistic levels (such as lexical, syntactic, or phonological). Lexical entrainment is understood as a generally observed tendency of communication partners to repeatedly refer to the same objects using the same terms (e.g., one partner refers to a presented shoe as “loafer,” the other partner adopts the term, and both partners keep referring to the object as “loafer” instead of “shoe”; see Brennan and Clark, 1996).

This emergent choice of lexical terms is remarkably stable within a conversation (and subsequent conversations) between two partners, and is at least partially partner-specific (Metzing and Brennan, 2003). According to Brennan and Clark (1996), lexical entrainment is based on the emergent forming of “conceptual pacts” between conversation partners, the implicit agreement on a shared conceptualization of an object for the conversational purpose. Similar processes also occur on the syntactic level, with syntactic structures of expressions made by one partner influencing (i.e., priming) syntactic structures of following expressions by the other partner (e.g., Pickering and Branigan, 1999; Branigan et al., 2000). Phonetic entrainment is conceptualized as the process by which partners in dialog adapt various phonetic parameters (such as their voice intensity, voice quality, and speaking rate) to each other and thereby start to “sound more similar” (e.g., Lee et al., 2010). Interactive alignment in communication is conceptualized as strategic (explicit) activity that is based on underlying automatic (implicit) processes occurring on different levels (see Pickering and Garrod, 2004; Garrod and Pickering, 2007).

In the context of entrainment in dance, it has to be emphasized that human communication has more dimensions than language, and that the interactive processes underlying alignment in communication also involve non-verbal communication channels such as gesture, body posture, and facial expression (e.g., Regenbogen et al., 2012; Levinson and Holler, 2014). According to Garrod and Pickering (2004), alignment between partners in dialog is reached in an interactive way via entrainment on all these levels, including the convergence of speaking manner and body posture. According to Levinson and Holler (2014), “human communication is a system of systems, where the burden of information can be shifted from one part to another.” The authors point out that “many speech acts seem universal in character, and so do the sequences of actions they construct – for example, pairs of questions, and answers, offers and acceptances, greetings and greetings, and so forth. These sequences, though typically expressed in language, are also embodied in other ways: a request (perhaps visual) may prompt a visible action, a wave another wave back, a passing of a needed item a reciprocal grasping and so forth” Levinson and Holler emphasize the predominant role of the gestural modality for depicting spatial relations, and point out that this has implications beyond the spatial domain, “for iconic gestures, and signs are well suited to depicting transitivity, and thus agents and patients.” They argue that gesture is therefore “especially well adapted to communicating about... visuo-spatial dimensions, which lie close to human preoccupations and are central to human cognition” On the basis of studies of language development in children, Levinson and Holler (2014) argue that the rhythm of conversation develops independent of and prior to spoken language, including the use of iconic gestures and turn-taking at rates that resemble conversations between adults, and is transiently slowed down again when complex language processing comes into play. With regard to the remarkable human capacities for rapid turn-taking and “sustained multi-modal deployment of vocal and visual signals of hands, face and body,” Levinson (2006) speaks of an “*interaction engine*” that is universal across cultures and pre-linguistic in nature, phylogenetically (it is claimed to have evolved in early *Homo* species long before any spoken language) as well as ontogenetically (it can already be observed in the “proto-conversation” of human infants at 6 months of age).

To summarize, entrainment is a complex phenomenon that seems fundamental to human activities on various levels, specifically in a social context. Even though entrainment can initially be understood as “coordination of rhythmic movement,” it is obvious that this definition is not sufficient for contexts such as communication, music, or dance. While, in emergent coordination, entrainment to an external pulse or between agents can occur automatically, based on implicit processes that do not come to awareness, entrainment in planned coordination typically involves explicit processes not instead of, but on top of the underlying implicit ones. In communication, strategic processes based on fundamental automatic ones contribute to successful alignment (Garrod and Pickering, 2007). In the context of dance, it is of particular interest that communication (including non-linguistic communication) can be viewed as a “particularly well-integrated form of joint action” (Garrod and Pickering, 2009), calling upon the proto-linguistic “interaction engine” (Levinson, 2006) as a basis for rhythm, gesture and turn-taking (Levinson and Holler, 2014). Specifically in dance or music, entrainment is often intentional (e.g., based on the intention to synchronize, see Knoblich and Sebanz, 2008) and may even have higher strategic goals serving to optimize artistic performance, which might require extensive practice and rehearsal. Entrainment in the given context has a spatiotemporal dimension rooted in bodily gestures (Leman, 2012) and, for the case of dance, in complex movement of the body in space (i.e., integrating representations of space). Furthermore, we assume that entrainment in dance, like in communication, might be partner-specific and even involve the forming of “conceptual pacts” (Brennan and Clark, 1996), and that it might have subjective dimensions that do not become visible to an observer but are experienced by and crucial for the entrained agents (Clayton, 2012; Leman, 2012).

### CONCEPTS OF ENTRAINMENT IN CONTEMPORARY DANCE AND IN *DUO*

In this section we outline the language for conceptualizing entrainment from the perspective of dance practice, using discourse from dance studies when possible to support our claims. Our aim is to enable a cross-disciplinary language, by sketching dancers’ insight about movement parsing, musicality, and partnering relationships, to inform studies of human entrainment across domains.

The term entrainment is not native to contexts of classical and contemporary dance. Dancers in classical and contemporary contexts describe synchronous dancing as dancing *together* or *in unison*. In classical contexts, group coordination in space and time is called *corps work*, as performed by the ensemble of the *corps de ballet*. As in *Swan Lake,* this may involve precise unison or complementary actions. *Connection* is a term used in some contemporary dance, martial arts, and swing dance contexts, to describe how well one feels togetherness with a partner. As described by Waterhouse (2010), the term *connection* has been named and cultivated in William Forsythe’s dance ensemble (hereafter referred to as The Forsythe Company) through master workshops in the Japanese martial art of Budo; in this context, connection is a basis for signaling involving tactile and visual cues. There is no universal term for complementary joint action in dance, aside from perhaps the description of dancing *musically*. The Forsythe Company uses the specific term *counterpoint* to define “a field of action in which the intermittent and irregular coincidence of attributes between organizational elements produces an ordered interplay” (Forsythe, 2009).

Should the language of entrainment emphasize coordinated movements, action, gestures, or movement? The terms motion, action, movement, and gesture are used distinctly among contemporary dance lineages, but little documentation exists to transfer these concepts (for an exception see Guest, 2005). Influenced by movement theorist Rudolf von Laban (see Sulcas, 1995; Caspersen, 2004), Forsythe writes about choreography and dance in terms of *motion* or *action*, not in terms of *gesture* (Forsythe, 1999, 2008). Dancers in The Forsythe Company also rarely use the term *gesture*, instead speaking about movement material and movement phrases. Neither the definition of gesture as limb motion related to communication (as in Hodgson, 2001) nor as limb movement that does not carry weight (Guest, 2005) is logical for *Duo*. Since the movement in *Duo* involves complex relations of limb movement and is abstract (not narrative), the authors avoid the term gesture and instead concentrate on the dance as an activity of motion. (Note that in this article the terms motion and movement are used synonymously.)

The proposal to understand entrainment via the operational definition of coordination of rhythmic movement implies the ability to resolve or parse motion. Parsing motion into elementary movements has already been shown to be challenging in situations of contemporary dance (DeLahunta and Barnard, 2005a,b). Often at issue is how to parse movements into sub-movements, and to understand how (or even whether) these subdivision relate to the dancers’ and choreographers’ intentions (e.g., Guest, 2005; Jola, 2007). The choreographic work of William Forsythe exhibits highly complex states of motion – involving complex torsions, polycentric initiation, and polyrhythmic layering of action (Kaiser, 1999; Caspersen, 2000, 2010). This can be viewed in Forsythe’s own body, in his lecture demonstrations and solo on the CD-ROM Improvisation Technologies (Forsythe, 1999). We emphasize that in situations of complex motion, one must have an interdisciplinary agreement about the appropriateness and methodology of motion parsing.

In brief, we propose to use aspects of German dancer, choreographer, movement theorist, and notator Rudolf Laban’s theory of movement and rhythm, to inform a body-specific framework for the study of entrainment, and analysis of entrainment in *Duo*. First we accept Laban’s approach to movement intention. Laban recognizes movement as a dynamic interplay of internal and external motivations, mixing voluntary and involuntary actions (see Hodgson, 2001). In this the body is framed as “not only an instrument of expression, but also an instrument of impression: there is a two-way traffic of sending and receiving” (Hodgson, 2001). Laban is well aware that this trafficking is not resolvable into single messages. Modeling the body as a channel of signals that are sent from brain to world and world to brain is not the approach taken here, nor advised.

Secondly, we borrow Laban’s understanding of rhythm in dance and human movement. Breaking from the *eurythmics* method of Jaques-Dalcroze (1913), Laban believed that dance was equal (not subordinate) to music, and that rhythm could emerge from the body as dance, without an acoustic source (Partsch-Bergsohn and Bergsohn, 2003). Hodgson (2001) describes Laban’s approach to rhythm as “alternation of opposite happenings” as “organized tension and relaxation, each with it’s own effort.” Note that this is a body-specific approach as to what rhythm feels like, as opposed to how to observe it. This anticipates that in a body-specific framework, the “more or less arbitrary definition of a reference point” (Clayton, 2012) for observing rhythmic processes can become less arbitrary through qualitative attention to the dancers’ experience, and, in particular, approaches to modeling that value, as Laban seems to, processes of work as energy conversion.

While rhythmic action to music features prominently in non-dancers’ (e.g., Holland, 2004; cf. Lepecki, 2006) expectations of dance, the diversity of choreographic approaches invites refinement of the perspective of dance as a response to an external musical signal (on Trisha Brown, see Phelan, 2004; on Merce Cunningham, see Copeland, 1983; on William Forsythe, see Vass-Rhee, 2010). Contemporary dance involves diverse musical environments, at times featuring interaction between dance and music, or dancers and musicians (see Vass-Rhee, 2010; De Keersmaeker and Cvejić, 2012). Even in the case of dancers performing rehearsed moves to recorded music with a beat, we take the position that the specific installation of the music in space (loudspeaker directions, volume, etc.), interaction with this via movement, and the social context, all influence how entrainment within the dance takes place. Thus we agree with Leman (2012) that entrainment in dance is of spatiotemporal, not purely temporal, nature, and that it should be considered in its ecological context. With this case study, we emphasize that the paradigm of dance as a visual art performed to an audio pulse should be used cautiously, as it is suspected that multimodal cues and feedback factor strongly in danced entrainment.

A last point is that the language of entrainment must address the “coupling” between dancers. In contemporary dance, coupling is commonly called *partnering* and usually involves touch. Duets, in classical ballet as well as in social dancing, may espouse having a leader or follower. In contemporary dance, as in *Duo*, these roles are not pre-determined nor fixed for the duration of the dance. We recognize that the negotiation of the roles of leading and following, even in forms of dance that name the learning and performance of these roles, reflects social values and subtle power negotiations (see Novack, 1990; Taylor, 1998; Manning, 2009). The concepts that Manning (2009) has theorized within her analysis of the tango as relational movement have influenced the framing of observables defined in this article (such as elasticity of tempo and cue intervals). Agreeing with Clayton (2012), we concur that dance is a “particularly good forum for investigating the interdependence between timing coordination and social power relationships.”

In summary, the approach taken here is to consider entrainment between dancers in *Duo* as coordinated interplay of motion and sound production by active agents (i.e., dancers) in the field, a definition that applies also to music and communication. Rhythm appears in the periodicity of these relations, and corresponds to energetic turning points in the dancers’ motions. We use the term movement and motion synonymously, avoiding the term gesture in respect of the dancers’ and choreographer’s own language for their motion. We emphasize that the coupling in *Duo* is non-hierarchical.

## *DUO* – A CASE STUDY OF ENTRAINMENT

When watching a contemporary choreography that appears to involve coordinated rhythmic motion, the task to study entrainment involves understanding multiple processes. How does entrainment appear? By which factors is it determined and influenced? What are the enabling constraints? How is it performed or created by the dancers?

As an example, consider the website *Synchronous Objects* developed for Forsythe’s group choreography *One Flat Thing, reproduced* (Forsythe, 2009; http://synchronousobjects.osu.edu/). The website reveals rhythmic activity that is much more complex than could be perceived by an observer in one viewing – making entrainment a matter of aesthetic perception. The website defines parameters (cues and alignments) that frame entrainment, while the influence of other factors (such as the environment of tables) remains backgrounded. From close review, it is obvious that the dancers do not need to be aware of the entire sum of coordinative relations in the choreography, but just need to know enough to accomplish their part. These preliminary observations suggests the value of directing entrainment studies in dance along three leading questions: (i) when and how is entrainment between dancers perceived by an observer in the audience, (ii) how is entrainment predetermined and planned by the choreographer, and (iii) how do the dancers create entrainment in performance? This article focuses on the latter, using the choreography of William Forsythe’s *Duo* as a case study. In the following we introduce the choreography, describe our methodological approach, and then highlight the perspective of performer Riley Watts.

### INTRODUCTION TO *DUO*

*Duo* is an approximately 10 min choreography for two dancers by choreographer William Forsythe, made in 1996 for Ballett Frankfurt. We report upon the reconstruction of the work by The Forsythe Company from 2012 to 2014. It has to be emphasized that *Duo*, like other pieces performed on a comparably high level of dance skill, has only been performed by very few dancers, and is recognized as requiring exceptional skills. Among the repertoire of William Forsythe, *Duo* is acknowledged among the dancers as a pristine exhibition of togetherness, requiring dancers to learn precise coordination of their movement in space and time. In addition to the artistic emphasis on synchronization, other characteristics that make *Duo* specifically valuable for the study of entrainment include that the choreography does not involve touch-based partnering, is practiced without music, and is coordinated by a breath score (i.e., the usage of the dancers’ breath and movement-based sounds such as footfalls; see Vass-Rhee, 2010). These features make it an interesting case of dance vocalization, described within the company as a form of conversation.

The literature on entrainment considers both emergent and planned coordination (see Phillips-Silver and Keller, 2012). In Forsythe’s work, the distinction between planned and emergent coordination varies, as illustrated by his development of techniques of improvisation (Forsythe, 1999). Notably, we have chosen *Duo* as a case study for the issue of entrainment precisely because it is a choreographed piece. This means that the dancers perform planned movement that is reproducible under conditions of rehearsal and performance, and also that *Duo* involves little material that is described by the dancers as improvisation.

The abstract movement in *Duo* is organized into sequences or phrases, some of which are named. In the case of the most recent cast of dancers (Riley Watts and Brigel Gjoka, see **Figure 1**), the dancers began rehearsals by learning and refining these sequences, with the help of William Forsythe, experienced dancers that had already learned or performed the piece, and videos of previous performances. To perform the duet, the performers had to cultivate a high level of awareness of what their partner was doing.

*Duo* involves fine control of gross motor movements. The movement is a contemporary development of the balletic practice of “*épaulement*,” a highly coordinated technique of creating spirals in the body (i.e., the body is in a dynamic state of torsion and counter-torsion, and this curvilinear motion brings the whole body into coordination; see Caspersen, 2010). The choreography modulates different degrees and kinds of movement complexity, in which polyrhythmic and polycentric coordination appear. Markedly, the dance also involves simple periodic motions performed in synchronization (e.g., running backwards, walking backwards while circling an arm, letting one arm swing like a pendulum).

The breath score of *Duo* involves inhalation and exhalation noises that are (within contextual variations) audible between performers as well as to the audience. The breath is made both through the nasal and mouth passages, affording texture, and tone-like character. Breath is, by its nature, periodic, involving the increase and decrease of tension that, according to Laban, is characteristic of rhythmic movement at large (see Hodgson, 2001). In creating correspondence between movement and breath, *Duo* capitalizes on a skill developed in infancy, namely that of extrapolating across sense modalities via structural likeness (Sklar, 2007). Feeling the body in motion while breathing, following the expansion and contraction of the rib cage, makes breath perhaps more “sensory” than we realize – just as perception often depends on action, when moving the head to direct gaze or extending the arm to touch with the hand. Breathing thereby intertwines voluntary action, action-based perception, and involuntary necessity.

### METHODOLOGICAL APPROACH AND OBSERVATIONS

Crucial aspects of entrainment in *Duo* have been established via discussion among the authors. In the following, we present our observations based upon the study of video recordings, annotation of these recordings, and insights from the perspective of performer Riley Watts. Video and annotation samples are provided online at http://www.dancelikething.org/Duo.html (video password: Frontiers).

#### Video material

The video sources (VS) spanned three separate performance of *Duo,* featuring two sets of dancers:

• VS1: Ballett Frankfurt, performed in Frankfurt, 03.09.2000; dancers: Jill Johnson and Allison Brown; musical score by Thom Willems;
• VS2: The Forsythe Company, performed in Darmstadt, 06.01.2013; dancers: Brigel Gjoka and Riley Watts; no musical score;
• VS3: The Forsythe Company, performed in Weimar, 09.12.2013; dancers: Brigel Gjoka and Riley Watts, musical score by Thom Willems.

#### Annotation

Based on Waterhouse’s prior work annotating data from the dancers in The Forsythe Company for the website *Synchronous Objects for One Flat Thing, reproduced* (Forsythe, 2009; http://synchronousobjects.osu.edu/), a simple system for the annotation of *Duo* was developed to create a time log of key features: namely modes of coupling, cues, and alignments. This system was used first to protocol excerpts of VS2, as well as to record questions and comments. The annotation was reviewed and developed by Riley Watts, at times consulting co-dancer Brigel Gjoka. Next, a rich target sequence featuring the diversity of modes of coupling was chosen and extracted from VS2 and VS3 (see supplementary material for excerpts and protocols). To emphasize material not addressed in these excerpts, a second target sequence was selected and annotated, showing the first minute of VS3 (this performance was preferred by the dancers to VS2).

#### Modes of coupling

Based on discussions with the *Duo* performers, four *modes of coupling* were defined and used for the annotation:

• *unison*: the dancers move together, performing identical movement at the same time;
• *complementary*: the dancers move together, performing different movements at the same time. The dancers describe these sections as being in counterpoint;
• *turn-taking*: one dancer moves and the other is paused. Note in the specific case of *Duo,* the paused dancer is in anticipation of a cue to join in, rather than waiting passively;
• *breaks*: both dancers collectively pause or rest.

Annotations of the *Duo* performances illustrate that the dancers’ interactions shift from mode to mode (i.e., in VS2 the longest unison section is 1′10″, the provided annotation illustrates faster shifting on the order of 2–10 s). Thus, not only had the dancers to attentively synchronize, take turns, pause, and complement each others’ movements, they also had to shift between modes with ease while knowing that they were doing so. The facility, coherence, and precision with which these forms of shared activity are entered and exited suggest that the dancers relied on a highly practiced set of skills, and a shared framework of their emergence. We suggest that the defined four modes of coupling can be considered varieties of entrainment framed by the choreography.

While dancing in unison, both performers of *Duo* contribute movement and breath in approximately equal balance. This can be understood as an example of synchronous chorusing without specified leader and follower roles, according to Phillips-Silver and Keller (2012) who state that “chorusing occurs when communicative signals (sounds or movements) produced by separate individuals make simultaneous and roughly equal contributions to the joint action as a whole.” As in mutual entrainment, the mirror-like affirmation of another body performing the same movement and producing breath simultaneously results in a feedback loop; as such it is rather mirroring than imitation with hierarchical roles.

Sections of predetermined turn-taking in *Duo* are significant, but short and relatively infrequent within the overall sequence. In these instances, the performers alternate producing movement and sound signals with little temporal overlap, which results in non-hierarchical and conversational turn-taking rather than in a hierarchical call-and-response structure. Such instances of aligned conversation-like interaction can be regarded a skilled activity of what Levinson calls the *interaction engine* (see Levinson, 2006; Levinson and Holler, 2014), as it involves joint rhythmic motor action and turn-taking on the basis of multimodal (i.e., visual and audible) signaling employing complex body movement, breath, and even voice.

#### Cues and alignments

The choreography of *Duo* prefigures changes in modes of coupling via *cues* and *alignments*. Cues and alignments have previously been defined, annotated, and visualized in the context of William Forsythe’s ensemble dance for seventeen performers, *One Flat Thing, reproduced* (*Synchronous Objects*). For the study of *Duo,* we used the following working definitions:

• C*ues* are predominantly planned (i.e., choreographed) aural and visual signals between the dancers that assist the coordination of their activity.
• *Alignments* are predetermined, short, choreographed instances of synchronization that only occur within complementary coordination.

In our annotation, we proceeded by simply cataloging the cues and alignments, using comments to develop discrepancies; a more refined approach with improved time resolution and subcategories of cues and alignments is in progress.

Cues differ in degree and kind. Cues in *Duo* are aural (breath cues, verbal cues in language, bodily sound cues such as stomping), and visual (both direct or peripheral). Although most cues are choreographic imperatives (i.e., directions from William Forsythe), some are unplanned, and might even be unconscious signaling by the dancers (e.g., head turning). In the case of *Duo*, cues may initiate motion (marking the transformation from stillness to activity) or happen within actions (e.g., while dancing, one performer audibly hits the floor as a signal and a dynamic change takes place). Lastly, cues reflect different formats of participation, in terms of leader/follower hierarchies. Cues can thereby be viewed as multimodal communicative signals (see Levinson and Holler, 2014) used implicitly and explicitly to coordinate joint action. For further discussion of cues, see section “Rich annotation of cues” where we compare cues in *Duo* to coordination smoothers, gestures such as exaggerated inhales or body sways that facilitate coordination between musicians but are not composed musical cues (see Phillips-Silver and Keller, 2012).

*Alignments* in *Duo* typically involve non-hierarchical collaboration. They may be static (e.g., arriving at the same pose as one’s partner) or dynamic (making the same movement together, such as flow, swing, or spring, etc). While the website *Synchronous Objects* judges alignments from the position of an outside observer, our approach of alignment prioritizes the dancers’ experience(s). The multimodality of cues and complexity of alignments makes clear that dancers in *Duo* tune to both auditive and visual feedback, and actively participate in choreographic contingencies.

### A PERFORMER’S PERSPECTIVE

The following section articulates the perspective of performer Riley Watts, who has been part of the most recent *Duo* cast (together with Brigel Gjoka).

#### Shared intentionality and temporal integrity

According to Watts, the choreographic structure of *Duo* consists of phrases where the dancers “come together” in unison (in which they dance the same movement synchronously) and then “separate” going “in and out of counterpoint” (performing different movements in more varied spatial and temporal relations than unison would normally allow). Distance is used here metaphorically; this means, that during “unison” the performers are not necessarily dancing spatially near to one another, and during “counterpoint” the performers are not necessarily dancing at a spatial distance (see **Figure 1**). Crucially, dancing together can be described as oscillation of modes of activity, with synchronization and chorusing being conceptualized as metaphorically close while complementary action is understood as rather separate.

Beyond displaying many sublime intervals of unison, the greater goal of the piece is to display real-time collaboration “on moving together in its many permutations, performing the art of *elastic temporal integrity*.” From this perspective, more important than the performers achieving “perfect” unison is how the dancers choose to play or engage time, using dynamic musical strategies based on their collaborative duet relationship. This perspective suggests that a definition of entrainment as an achievement of synchronization, in the sense of sharing and producing identical movement simultaneously, did not always correspond to the goals of *Duo*, in both the choreographer’s and the dancers’ intention. More than just sharing the “intention to synchronize” (Knoblich and Sebanz, 2008), the dancers work toward the (more complex) shared “goal of communicating with each other” (Garrod and Pickering, 2009). With regard to the versions of *Duo* performed with a musical score by Thom Willems, pianist David Morrow has acknowledged that his role as a musician “required sensitivity not to disrupt the conversation they (the dancers) are having,” affirming that *Duo* is conversational in nature.

**FIGURE 2:**
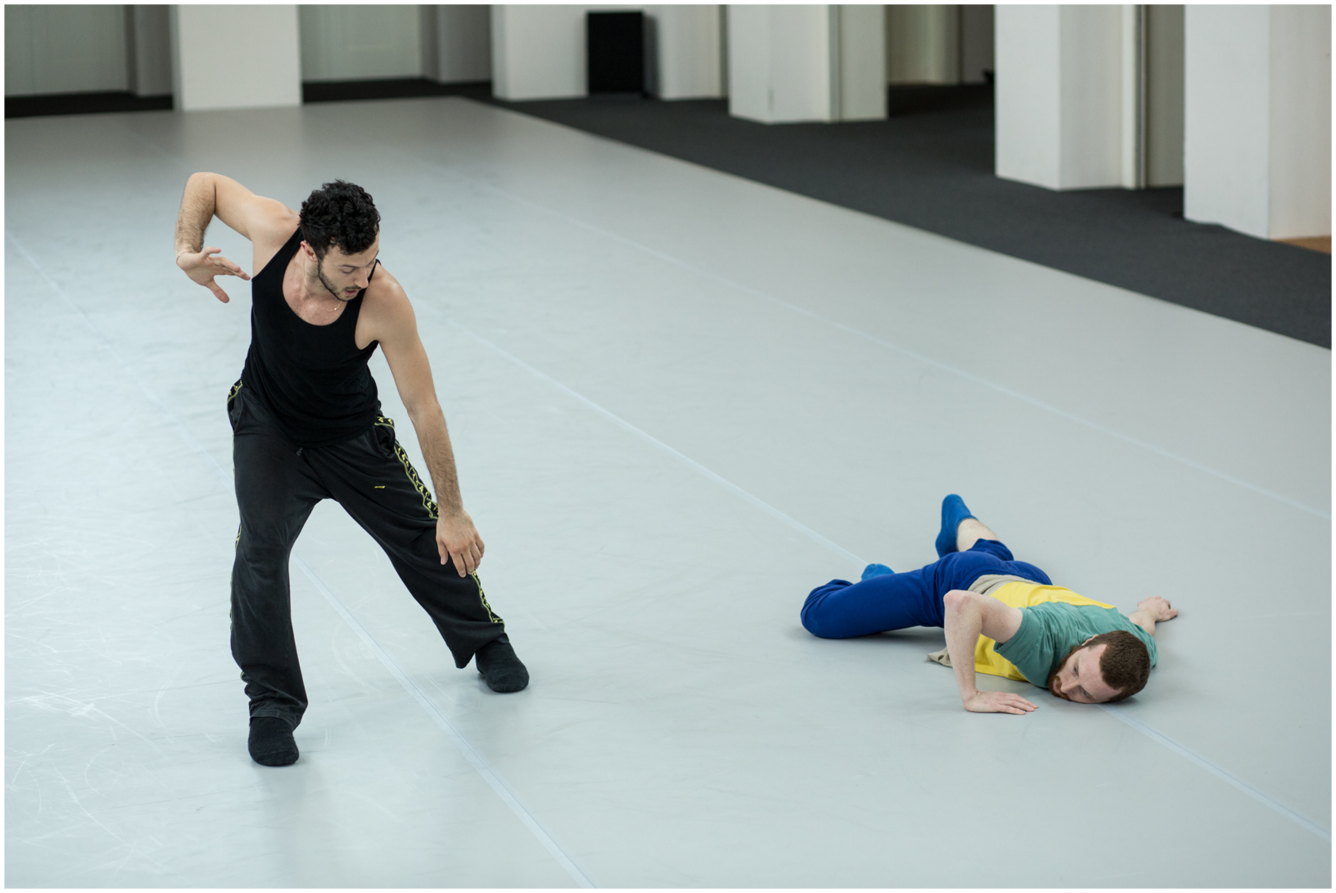
**Brigel Gjoka (left) and Riley Watts (right) during a rehearsal of *Duo* in Dresden in 2012.** According to Watts, the picture captures “a moment of ‘contrapuntal’ surprise.” “Brigel decides to deviate and to not go to the floor as we’ve learned choreographically, and as I chose to do. When I am looking at him in the photo it is just after I have realized that he has done something ‘surprising’ and I am waiting to see how we will ‘reconnect’ after this moment of separation from the material. It has the expectation of unison that does not become fulfilled and is right before the moment where we re-entrain with the material. You can see by our right elbows that we are still in the same place choreographically.” Photograph by Dominik Mentzos.

The expertly mutual entrainment in *Duo* is, surprisingly, not achieved in a pedantic fashion of staying on-beat. As a crucial aspect of performing together, attempting to dance only with the identical tempo and rhythm as one’s partner would remove the elastic temporal integrity of *Duo*. Particularly in the Watts/Gjoka version of *Duo*, the dancers intend to be sufficiently in-tune to one another, so that they are able to perform *Duo* as a well-aligned dialog (as defined in Garrod and Pickering, 2009). Thereby, the piece’s timing, based on the practiced choreographic structure and movement phrases, can be adapted spontaneously during the performance by the dancers as they react to each other. To accomplish this, each dancer is responsible for intentionally deviating (via slowing down, speeding up, etc.) from an even tempo. This is done in order to surprise the other, knowing that the surprises will ideally elicit a reaction from one’s attentive partner. **Figure 2** illustrates this interaction by showing “a moment of ‘contrapuntal’ surprise,” as Watts remarks, initiated by a deviating action by Gjoka. In that way the piece’s “temporal momentum” gets built from a series of individual choices as the dancers “play” or “push” each other forward in game-like musicality. Watts remarks: “perhaps at some point when we became really fluent in this piece, when a lot of the sensory activities began to happen naturally, automatically, we needed the ‘surprise’ moments to jolt our predictive abilities back into unknowing.... When Brigel surprises me with a musical or spatial anomaly, it is actually for the purpose of introducing a cognitive anomaly from the series of actions in which I am already fluent. Maybe in that way, entrainment is used like the groundwork or pathway for us to communicate through learned cognitive patterns and anomalies in a performance setting.”

In this regard, dancing *Duo* can be seen as a multi-layered instance of a non-linguistic dialog (and thereby a “particularly well-integrated form of joint action,” according to Garrod and Pickering, 2009) based on shared intention and involving rehearsals during which a common representational ground is achieved via implicit and explicit processes: the experience of a shared context and dancing together, and linguistic dialog that focuses on exchanging experiences and framing goals. Crucially, entrainment in *Duo* also involves joint attention toward the choreography as well as mutual attention toward the duet partner and the dancer’s own body. While not observing the same external object, the dancers work on “experiencing the same thing at the same time,” knowing that they are doing this together, thus building up a rich shared representation that can serve as common ground for performance.

#### The rehearsal process

From the dancers’ perspective, the performance of *Duo* represents an event of collaboration achieved by rehearsal. As in musical rehearsal, *Duo* is learned by movement-based exchanges, as well as linguistic dialog on both subjective experience of the duet action and framing of collective goals within the parameters of the choreography.

In particular, the chorusing in *Duo* is cultivated through rehearsal. From experienced-based associations between sensorimotor processes, a shared embodied representation of the choreography is established. To learn the movement phrases, dancers began by observing and imitating movement from videos and live demonstration by the rehearsal director and other dancers with previous experience of performing *Duo*. Visual mimicry of the movement, though necessary in the learning process, was not the only priority in reproducing the choreography with integrity and skill. Instead, the dancers had to “translate” the observed movements into the “language” of their own bodies with the help of verbal descriptions from their partner, the rehearsal director and previous *Duo* performers who assisted as teachers. This suggests that individualized mechanics and attention to sensation are important to the process of learning, rehearsing and staging *Duo*. The alternation between demonstration, detailed verbal translation of sensorimotor experience, and action reproduction was a typical format for the learning process of this piece. From the performer’s individual perspective, the descriptive dialog of sensory details was essential to the learning process to such a degree that it would eventually inform the visual appearance of the movement and even the signature movement quality specific to each couple.

It can therefore be claimed that *Duo* dancers during the rehearsal process build up “conceptual pacts” comparable to those conceptualized in the context of linguistic dialog (Brennan and Clark, 1996; Metzing and Brennan, 2003). Compared to these examples, conceptual pacts between *Duo* dancers are likely to take longer to be established and have to be more consistent over longer periods of time, as they are needed as common ground for rehearsals and performances in the long run. They are also likely to be more complex in content (i.e., including more complex concepts) than those in conversation, as they do not refer to representations of external objects, but their object is the multimodal representation of the dance as such (or parts of it). Finally, such “conceptual performance pacts” in *Duo* can afford to be highly partner-specific, as partners do not change (as in conversation); indeed, the specific characteristics of individual casts of highly skilled dancers are likely to be crucial for the artistic value of the performance.

In rehearsal, it was agreed that imitation and knowledge of the appearance of “body form” (i.e., the shape that becomes visible to the audience) was neither the subject of the work nor the ideal method to learn. Though each dancer approached the learning process slightly differently, the dancers agreed that only copying the shapes of the movement would not enable performing this piece “well.” Rather, what was advised was to focus on learning the details of one’s sensation, and its relation to the quality or form of the movement; this makes learning to dance *Duo* a process of attention to sensations that the dancers are experiencing simultaneously. Skin sensation, or intensity, was one focus of attention used. As an example, Watts provides “When I am extending my arm behind me there is a particular sensation of the skin stretching across my chest and down my arm to my hand. I could show you this movement and you could copy it easily, but without you paying attention to the sensation of stretching that I described, we both would be experiencing something slightly different.”

This suggests that in *Duo*, as with much of the work in The Forsythe Company, movement is achieved in relation to a cultivated attention to what an action feels like rather than purely what it looks like, and that such attention might be helpful for constructing rich embodied representations as basis for anticipatory imagery. Neither expression-based, nor affect-based, movement has to do with cues of bodily sensation: modulations of tension, skin intensity, joint torsions etc. Moreover, the dancers in The Forsythe Company take time in rehearsal to verbally converse about these aspects of dancing, actively searching for shared representations to build up conceptual common ground. Such cultivated and trained movement imagery seems to be an essential tool that strengthens both predictive abilities for cueing and the feeling of immaterial connection between the performers. When questioned whether connection could be achieved with an absolute stranger, Watts suggests that in the case of *Duo*, two dancers could not dance the piece unless they had achieved some level of sensorimotor training, presumably requiring prior dance experience similar to that of The Forsythe Company.

#### Breath and the experience of connection

Watts described the use of breath in *Duo* as a “song-like” description of the motion that helped him to remember the complex sequences of choreography. It also facilitated staying in time during the performance; both in achieving consistent timings of dance phrases, and having an agreed tempo from which to vary with one’s partner. The sounds emitted from inhales and exhales provide valuable information, helping the dancers to keep track of each other, a bit like echolocation. Watts described the use of breath in *Duo* as “a communicative extension of somatic motion, ideally perceived as relating directly from actions seen to actions heard.” In the beginning of the piece, the dancers’ attention to their individual and collective breath seemed to be more deliberate and prioritized than later on, when their bodies were more fatigued. To the dancers, the breath song can represent a modified linguistic tool in order to stay “in conversation” with each other and to indicate cues (compare Levinson and Holler, 2014).

In order to understand the details of breath and cue usage, we refer to a selected example from the opening moment of *Duo* (VS3, available online as the “Beginning” clip). Watts initiates the first movement by giving Gjoka a breath cue in the form of an audible exhale. Watts and Gjoka are at a far distance from each other on stage, which affects their ability to connect. Watts states: “I know he is listening to my cue, and from a distance I have to feel that he is ready to begin. It is our job to imagine the connection between us and sometimes I would imagine a phantom limb connecting us between our shoulders. I try to imagine, what does Brigel feel like right now? What does his body look like? Can I feel what his body feels like? Only when I feel that we are sufficiently connected do I audibly exhale and begin the first movement.” This mental image as mode of connecting came from Watts’ imagination developed during the rehearsal process, not from the choreographer’s instructions, as a strategy to connect to his partner. As this quote indicates, *connection* is a term often used by the dancers to describe a quality necessary for their time keeping. This subjective experience of connection might refer to dynamic features of the shared embodied representation of the piece, consciously accessible via imagery.

In our conversations, it was clear that the quality of connection between dancers in *Duo* was not only variable, but highly considered. Once experience had been gained, nervousness was cited as a block to connection and entrainment, as in the first performance of Watts and Gjoka (VS2) where the dancers felt that in moments they were not as well connected as they could have been. The dancers’ personalities and duet “chemistry” (potentially resulting in “conceptual performance pacts,” see section “The rehearsal process”) also appeared to be an important factor, suggesting that individual duet pairs develop their own specific styles of entrainment, as kinematic signatures, comparable to styles of play in sports or in music.

## ENTRAINMENT AS LEARNED, REHEARSED PRACTICE

For the case of contemporary dance, entrainment as a spatiotemporal phenomenon can be described as a form of subjectivity requiring not only the ability to sense and produce rhythm, but also a process of integration that enables an apparent temporary continuity and accumulation of experience. In both pulse and non-pulse based entrainment, humans must select information to predict and participate in events (Schachner, 2013). This has been studied in the comparatively simpler cases of sensorimotor synchronization of tapping with periodic actions and referents (Repp, 2005), as well as in more complex types of action (Repp and Su, 2013; Demos et al., 2014). In complex (inter-)action, such as dance, this prioritization is hardly possible without experience or feedback, and is therefore based on memory and learning.

Leman (2012) argues that entrainment between musicians can happen quickly or spontaneously only after long-term practice, meaning that for humans it is a learned behavior built upon innate neural capacities. Furthermore, affective components come into focus with dancers who train and develop choreography together (see Phillips-Silver and Keller, 2012). The ability to entrain socially, as we have come to understand it within The Forsythe Company, seems to rely on longstanding rehearsal processes, involving training in which haptic and proprioceptive feedback and techniques of gaze usage and sound production are used to develop the dancers’ awareness of their own and others’ movement for the purpose of better decision-making during performance. Practiced skills include the ability to causally situate oneself in time and space, to remember details of danced experience, to modulate attention, turn-taking, as well as the ability to judge oneself and to accept being judged. This approach reflects Forsythe’s fascination with the human body, which he describes as “wholly designed to persistently *read* every signal from its environment” (Forsythe, 2008).

The practice of social entrainment in The Forsythe Company is supported by the more intimate practice of duet work, as mutual entrainment. Dancers in *Duo* practice in quiet conditions (typically with low background noise and without music) so that they can both better hear each other work, as well as take agency in the learning process to initiate dialog about what they are doing. The training-based sensitization of the dancers in The Forsythe Company to communally attune to signals supports their ability to entrain despite distractions, actively ignoring distracting stimuli, and the ability to judge or enjoy entrainment. In staged performance, the dancers’ motivation to listen and connect, when taken within the high-risk environment of a large space and large audience, and the low thresholds (quiet conditions, black background, absence of visual stimuli), are factors that enable entrainment.

Despite the common expectation that visually understanding form, which is supported by training with mirrors, is essential to learning in dance, the attention to bodily sensations in *Duo* is multimodal in nature. The dancers in The Forsythe Company must divide their attention between awareness of the body-in-action (i.e., integrated proprioceptive, kinesthetic, haptic, and vestibular stimuli), features of the spatiotemporal field including visual and audible cues, and cognitive real-time evaluation of what has happened, how well it is happening, and what should happen next. The focus on multimodal cueing in the ensemble practice implies, correctly, that imitation and mimicry are skills within a much larger array of aptitudes. We predict that, in *Duo*, and more generally in contemporary dance, learning to shift attention to signals other than the mirrored image of the dancer’s own body, and to do so in rhythm with others, corresponds to stronger entrainment in staged performance.

In their discussion of musical rehearsal and ensemble musicians, Phillips-Silver and Keller (2012) highlight three skills necessary for joint action and entrainment: adaptive timing, prioritized integrative attending, and anticipatory imagery. We conclude our discussion of learning in *Duo* by highlighting how these skills appear in our case study. In *Duo*, adaptive timing involves cue and alignment negotiation, as well as an at-large agreement to share (or take responsibility) to push and play with timing, via inflections of normative movement tempo and dynamic. Spatiotemporal entrainment in *Duo* therefore relates to the dancers’ ability to share the intention of regulating spatiotemporal actions within the context of performance. Adaptive timing choices in *Duo* also seem to coincide with Laban’s movement effort categories, developed to describe “work-study” methods of factory workers. Hodgson (2001) describes Laban’s effort as “(i) that which strives (contends) or fights, (ii) that which plays with … something, which does not necessarily involve exertion.” Similarly, Watts compares his approach to time in *Duo* as “pushing the other,” not “waiting” for one’s partner or “gaming” and “surprising the other in time.” Laban’s effort categories might thus be useful parameters for understanding the affective layers of entrainment, as aspects of adaptive timing.

When comparing performances by different dancers (or “casts”), we found that typical timing choices of individual casts were recognizable, almost as signatures. This observation might point toward representations of spatiotemporal and specifically dynamical movement characteristics as one crucial aspect of the proposed “conceptual performance pacts” between *Duo* partners. Furthermore, the dancers in *Duo* constantly shift their attention between peripheral and direct vision, sensations of tension and countertension, feeling of bounce, floor contact, sound and feeling of their own breathing, and external auditive information (partner, audience, etc). This implies that dancers in *Duo* share with musicians the ability of prioritized integrative attending: simultaneous attention to one’s own actions, others’ actions, the aggregate structure that results from coordinative actions, and the ability to vary which aspect of this information is of high or low priority (Phillips-Silver and Keller, 2012).

While it is difficult to assess whether dancers have precisely identical shared representations of an “ideal movement,” the performers’ experience shows that individually refining representations while collectively conferring is an active part of the *Duo* learning process. Clearly, the operating framework of *Duo* involves flexible musical phrasing to the danced material, requiring a common ground of understanding the movement phrases. One metric schema that might facilitate this is the “breath song,” described by Watts as an internal timekeeper, through which variation can be developed. Rather than anticipating breath, it is likely that in *Duo* much of the anticipatory imagery (Phillips-Silver and Keller, 2012) is based on a mixture of visual and proprioceptive feedback. During the learning process, Forsythe directed the dancers to visually observe the curvature in their arms as extended kinesthetic descriptions of the posture of their bodies in movement, thus relating limb action to gross motor action via visual feedback. They were also directed to *observe the sensation* (by means of all available sensory modalities – kinesthetic, proprioceptive, tactile, auditive, and visual, as well as multimodal imagery of body and movement) of counter-tension between the shoulders hips, spine, and lower legs, as they engaged in complex actions of winding and unwinding. Watts states that “when I am ‘listening’ to my body or feeling ‘texture’ to my dancing, I am performing more consciously.” Dancers practiced achieving unique states of disequilibrium, by following the advice not to let one’s pelvis rest in equilibrium, but to dance with it intentionally misaligned in order to produce a genuine falling motion when traveling through space. The kinesthetic image to dance with buoyancy in one’s feet was also very important to enhance the desired musicality.

Monitoring one’s position in space, via cues of falling and floating, are important to *Duo*. Impact cues, such as tapping a finger, moving one’s feet, or bobbing one’s head (see Repp, 2005; Schachner, 2013) might be extended to the dancers’ ability to feel coordination in the gravitational field. Rather than synchronizing impacts, the ability to coordinate anticipatory up-beats or springs at the height of a jump, is presumably an expert skill in dance that could motivate further studies. As is often said of conductor Leonard Bernstein, “there are no downbeats, only upbeats!”

## MEANINGFUL MEASUREMENT OF ENTRAINMENT IN *DUO*

How can entrainment in *Duo* be scientifically investigated and measured? We have started by outlining differences in concepts (and hence the use of language) relevant to the interdisciplinary study of entrainment. We then have reported our case study of entrainment in Forsythe’s choreography *Duo*, including annotations of video material from different performances and insights from the personal perspective of *Duo* performer Riley Watts. We will conclude this article by suggesting explicitly how further studies could be conducted and what questions of scientific and artistic interest they could focus on.

### COLLECTING DATA

In our case study, video recordings of *Duo* performances were used for annotation and as basis for discussions. Naturally, these recordings were not acquired under controlled conditions, and performances differed in various uncontrolled (and uncontrollable) ways. One benefit was that these examples were valued by the artists, and thus believed to have rich content. A limitation is the video resolution (note, the video examples provided online are of lower resolution than the full data files used for annotations).

Alternatively, the dancers’ performance could be recorded in a laboratory setting using a variety of devices such as video cameras, motion capture, electromyography, wearable sensors, and microphones. Such settings could provide meaningful data for specific research questions. Yet, following the comments of dancers after extensively experiencing dance in laboratory settings, it is questionable if the practice of entrainment in *Duo*, as it has been realized by the performing dancers and appears to be a fundamental part of the performance, could be achieved in a non-theatrical setting. Specifically, conditions that directly influence or interfere with the dancers’ bodily perception (e.g., marker-based motion capture) might preclude entrainment as it is achieved in the studio or in a theatrical setting. Approaches that require such methods of data collection (such as topographical gesture analysis, see Naveda and Leman, 2010, or principal component analysis based on full body motion capture, see Toiviainen et al., 2010) are therefore not recommendable for studying entrainment in *Duo*. Instead, we suggest to use video sources from real-world dance scenarios (i.e., performances or stage rehearsals), as well as other types of recordings (e.g., sound), that do not interfere with the dancers’ perception and (inter-)action.

### PROPOSED METHODOLOGY AND HYPOTHESES

As indicated in the introductory section, scientific measurement of entrainment in *Duo* requires a quantifiable working definition of synchronization. We have demonstrated that synchronization in *Duo* does not involve moving “in time with an auditory pulse” (Schachner, 2013). Rather, like musicians, the dancers simultaneously coordinate the production of movement and sound between them. By eye (and ear), it does appear that *Duo* involves a beat or pulse that is somehow “internal” to the dance. Using established methods of data analysis and video data with proper resolution, the timekeeping properties of *Duo* could be examined as follows.

A possible empirical approach could be similar to that used in studies with jazz musicians (Doffman, 2008, 2011) or birds (Patel et al., 2009; Schachner et al., 2009; Schachner, 2010). Inter-onset intervals in the audio signal (i.e., onset of breath noises) could be used to generate a series of time events, resulting in an ambient audio rhythm. Although the recordings do not enable separation of the individual dancers’ breath, a composite duet pulse can be resolved from pitch onset. (Assigning single breath signals to individual dancers, for example by dancers wearing microphones, would increase the amount of achievable information, e.g., about the use of breath cues). A motion pulse could be generated on the basis of head positions and limb movement onsets, via frame-wise coding of head and limb positions relative to a body reference from given video sources. Bounce could be approximated from head and body signals, (e.g., by calculating the frequency spectrum via Fourier transform). Similarly, limb movement parameters, such as turning points of swings, rebounds, foot falls, or twists, could be decomposed to produce key frequencies for each dancer, and phase relations between dancers. We stress that visual markers of endpoints in simple harmonic motions (such as winding and unwinding, or swinging a limb) are meaningful observables, as they relate to turning points of energy; this helps to relate the spatiotemporal measure of entrainment to the Laban perspective of rhythm as relying on effort or work.

Using well-established methods, the following hypotheses could be tested:

(i) We predict that the rehearsal of movement in *Duo* sets a movement pulse that, while subjected to inflection, is consistent across performance trials by the same cast. We do not expect this pulse to have a consistent tempo across the entire dance; rather, each phrase of movement appears to have its own normalized tempo. When observing our video sources (specifically, comparing VS2 and VS3), we found that the performances often appeared to sync-up, despite key inflections of tempi.
(ii) From Watts’ description of the relation between movement and sound production, we predict the audio pulse to be relatively analog to the movement pulse, especially during unison sections. We predict that hierarchical or polyrhythmic relationships between movement and audio signals will be more common in complementary action.
(iii) We expect the measured relative phase-angle for movement time data to be smaller for unison and larger for complementary coupling, meaning that the dancers’ movement is more synchronous in sections where they do the same movement than when they relate different movements. We predict hierarchical or polyrhythmic relations to occur more often in complementary coordination than in passages of unison.
(iv) From discussion with Watts, we expect there to be a threshold of phase perturbation for entrainment, meaning when phase angles are too high, then the dancers would experience a loss of connection. However, based upon our initial video analysis and discussions, we predict that the dancers’ assessment of entrainment will not correspond purely to statistical synchronization of movement onset. Rather, we expect that experienced “strong” entrainment will correspond to more frequent and/or shared acceleration of the movement pulse, and that entrainment experienced as “weak” will involve less frequent and/or unshared acceleration of the movement pulse (strong and weak entrainment are specific terms addressed in section “Dancers’ assessment of entrainment”).
(v) From our study of two video records of different casts of dancers (VS1 vs. VS2 and VS3), we hypothesize that each pair of dancers performing *Duo* develops a unique timing signature. Using the layered approach described below, it could be studied to what extent these aspects are choreographed or personal variations in time attitudes.

### A LAYERED APPROACH TO STUDYING ENTRAINMENT IN *DUO*

To investigate *how* dancers doing *Duo entrain*, engaging in the *activity of rhythmic coordination of motion and sound in the frame of a choreography*, a layered approach is advised. In this section, we develop a framework for a body-specific layered model of entrainment, involving quantitative assessment of timings to qualitative assessment from the dancers. This would require linking timing information systematically to the annotation of cues and alignments and to dancers’ assessment and judgment of their performance. By linking results of the analysis of audio and movement signals (period, phase relations, etc.) with the dancers’ assessment, we expect to find relations between measurable signals and entrainment.

#### Rich annotation of cues

To understand entrainment in *Duo,* a more detailed taxonomy of cues and alignments would be needed than used previously in *Synchronous Objects* or provided in the reference annotation. In *Duo,* cues operate on the common ground of shared movement and perceptual goals; as such, they are instances where timing choices are pre-agreed to be achieved. Cues operate both on an action level for the dancers as well as on a choreographic level, creating timing expectations for the audience. These aspects are worth studying in the context of entrainment.

We propose to conceptualize cues as nodes, characterized by three factors: the interval, the coupling hierarchy, and the sense modality. The first factor, the interval, refers to the temporal duration between the signal and the desired resulting action; this is indeed a matter of reaction time, but also of choreographic design. For the audience, longer intervals are likely to create expectation or suspense and might therefore be experienced as causal cues, whereas shorter intervals might remain unnoticed (masked cues) but nevertheless create surprise when change appears. For the performers, this also translates to different physical experiences: on one hand is the surprising or startling cue without preparation, on the other hand is the prepared cue creating active anticipation and adaptation. The second factor to annotate is the coupling hierarchy. This refers to the dancers’ roles as leader and follower regarding action initiation and decision making, or the absence of such roles; in the latter case, cues are open to real-time negotiation. The third factor, the sense modality, refers to the mode of information that is predominantly used for signaling and receiving feedback. We propose that the dancers’ experience of working with cues might be essential to their ability to entrain, and to make their entrainment recognizable for the audience. We predict that cue characteristics, defined by the three described factors, influence the spectators’ perception of entrainment, and expect that shorter, non-hierarchical cues are likely to evoke surprise, whereas longer hierarchical cues rather create casual satisfaction of comprehension.

A question arising from these considerations is whether cues in *Duo*, and specifically aural cues, can function as *coordination smoothers*. Phillips-Silver and Keller (2012) define coordination smoothers as actions or modifications of behavior that deliberately create predictability, and thus facilitate coordination. In music, coordination smoothers include, exaggerated movements associated with breathing, body, sway, and ancillary performance gestures, such as head nods. According to the authors, this implies that coordination smoothers appear to be ancillary cues or entrainment modes, and that they are often unintentional, but emerge from field-specific valuing (prioritizing) of certain cues over others *in the composition*. In the case of William Forsythe’s work, the attention to aural and visual composition occurs simultaneously, making identification of coordination smoothers difficult. Dancers nevertheless seem to use auditory feedback to reinforce movement cues, similar to musicians using gesture to reinforce aural cues. These aural cues, though not ancillary to the paradigms of Forsythe’s choreography, have a certain quality of musicality; they are integral to the composition of the work. We observe that the performance of entrainment in *Duo* is clearly multimodal in nature, and much of the perceptual prowess in The Forsythe Company is exceptionally hybrid, involving the interplay of attention to and feedback from skin sensation, breath sensation, visceral awareness, and acoustic and visual information. It can therefore be expected that cues and coordination smoothers are also potentially bi- or multimodal in nature, especially if they arise in an emergent manner from the dancers’ rehearsed interaction.

#### Dancers’ assessment of entrainment

Doffman (2008) has shown that in the context of Jazz, “the link between the musicians’ perceptions of their timing together and what appeared to be happening in the timing data varied.” This occurs despite timekeeping being, as it is in *Duo*, an honored, discussed, and fundamental aspect of the artistic work. We have investigated the possibility of finding parameters for understanding entrainment by asking Watts to define *strong* vs. *weak entrainment*. Watts described strong entrainment as when the dancers *react* to each other’s timing of initiating movement through cues and spatial alignments, and weak entrainment as when the dancers are either too distracted and/or cognitively overloaded with the choreography to be *reactive*. Weak entrainment could also correspond to lack of focus and missed cues. This suggests that tempo inflection (i.e., acceleration) as a form of reactivity, might be a parameter for assessing entrainment in *Duo*. Also important would be to know to what extent the dancers’ assessment is linked to quantitative features in the time data.

### A PROPOSED MODEL OF ELASTICITY

In section “Shared intentionality and temporal integrity” Watts emphasized that what is important in *Duo* is *how* the dancers choose to play or engage with time. Watts describes this real-time collaboration as “performing the art of elastic temporal integrity.” To conclude our discussion of entrainment in *Duo,* we propose a model of entrainment that is motivated by our case study and neighboring concerns from analyzing entrainment in Jazz. Doffman (2008) confirms that in a jazz setting, “there is an understanding that a degree of give and take is necessary for the music to be expressive,” also that jazz musicians “often speak of a certain elasticity in the timing between players for the music to work.” Regarding one of his data sets, he remarks that the “entrainment between these players is sufficiently elastic to allow for considerable expressivity in the relationship.” Can this elasticity be quantified?

We argue that it can, and that simple second order analysis of linear phase plots might reveal valuable information about the *how, how much*, and *in what case* of entrainment. According to the proposed model of elasticity, entrainment involves frequency and quality of interplay, measurable in the data phase space. This means that in the case of *Duo,* inflection of the audio and movement pulse is desired, as shared accelerations and decelerations. We suspect that expert *Duo* dancers’ skilled pulling and pushing of time would appear differently on such graphs than novices’, potentially revealing a difference between “pliancy” and “nuance” of skilled entrainment, and “snapping” or “broken” connection, corresponding to inattention and mistake. This is also an issue of duration (in that a good “elastic” maintains elasticity for a long time) and frequency (expert entrainment might involve more frequent and more complex collaborations on pulse relations, compared to novices). These second order effects can be computed from the linear phase plots of relative phase over time. We understand that the physical metaphor of elasticity is different from second order analysis of the relative phase data. Pragmatically, we recognize that the metaphor can be useful as it is already used in the working language of artists performing *Duo*, in Doffman’s interviews with jazz musicians, and an aspect of philosopher Erin Manning’s (2009) analysis of relational movement. While we do not expect human entrainment (in dance or at large) to always be elastic, we believe that the notion of elasticity in assessing human entrainment can be applied to diverse contexts, and is one example of how the “interplay” or “tendency” of synchronization can be meaningfully quantified in studies of entrainment.

## CONCLUSION

Based on William Forsythe’s choreography *Duo* used as a case study, we have emphasized the importance of using field-valued works of contemporary dance for the interdisciplinary study of entrainment. We have argued that this instance of skilled performance in contemporary dance is strongly based on entrainment between the dancers, and does not require music or an external rhythm or beat. Thus, while *Duo* challenges the definition of entrainment in dance as the coordinated response to an external musical signal, it supports the existing definition of entrainment as the coordinated interplay of motion and sound production by active agents (i.e., dancers) in the field. Additionally, entrainment in *Duo* involves automatic and strategic processes of communicational alignment. In this regard, *Duo* can be seen as a multi-layered non-linguistic dialog based on the common ground of shared intentions and multimodal representations.

The approach to entrainment proposed in this article provides many opportunities to understand how the dancers who have learned to dance *Duo* understand their own skill set for entraining. We have shown that entrainment in *Duo* requires a longstanding rehearsal process, involving training in which the dancers’ awareness is developed for clear decision-making, and resulting in the forming of “conceptual performance pacts” between duet partners. A strong intrinsic motivation to listen and connect to each other via multimodal cues and feedback are essential. We suggest that this example of *expert entrainment* is a skilful perceptual activity of rhythmic collaboration based on sensorimotor knowledge. The case study demonstrates that skill-based entrainment in dance requires the integration of implicit and explicit processes, as well as their automatization via practice. We argue that a body-specific framework for discussing human entrainment across domains should consider entrainment as a complex process based on integration of multimodal signals, with attention to compositional prioritizing and coupling hierarchies. Entrainment in contemporary dance should therefore be studied on multiple levels.

From this interdisciplinary case study, we hypothesize that while entrainment in *Duo* is achieved without training to an external pulse, it manifests pulse-based movement and sounds. We suggest that *strong entrainment* between dancers in *Duo* does not emerge as “perfect” unison (i.e., perfect-beat synchronization between partners), but rather as shared elasticity, pushing/yielding to the temporal pulse. Rather than modeling entrainment purely in terms of relative phase relations, we propose that a model of entrainment in *Duo* should take into account the elasticity of timing coordination. We thereby demonstrate how the scenario of two skilled dancers synchronizing and de-synchronizing their movements while dancing choreography together in the absence of an external rhythmic signal can be applied as a conceptual benchmark for the understanding of entrainment.

## Conflict of Interest Statement

The authors declare that the research was conducted in the absence of any commercial or financial relationships that could be construed as a potential conflict of interest.
