# Supplementary material for: Doing Duo – a case study of entrainment in William Forsythe’s choreography “Duo”
Source: Front Hum Neurosci. 2014 Oct 21;8:812. doi: 10.3389/fnhum.2014.00812 (PMC4204438; doi:10.3389/fnhum.2014.00812)
Supplement: Supplementary file 1 [file Data_Sheet_1.PDF]

Annotation Examples
last edit: May 25, 2014

Key:
U = unison
C = counterpoint
TT = turn taking
B = break/rest
AL = alignment
cue = cue Name*
***Name of person responsible for cue leading**
CS = coordinative smoother
< = comment
RW = Riley Watts, EW = Elizabeth Waterhouse, BB = Bettina Blaesing
timed=*approximate time in seconds on the Darmstadt video clip,*
approximate because of quicktime resolution of +/-5 seconds
timew=approximate time in seconds on the Weimar video clip,
approximate because of quicktime resolution of +/-5 seconds

Darmstadt and Weimar Sample Clip
AL (timed=0) (timew=0)
<EW dynamic alignment in movement, seems to be a starting impulse for the next sequence in unison
<RW this is that moment of being not-so-together musically and then coming back to the checkpoints. unintentional counterpoint within unison that happens either due to lack of concentration onto your partner or as a choreographic choice of the dancer
U (timed=0-13)
<EW darmstadt and weimar video are different here, change of choreography, note how in darmstadt video how pulse-like the breathing sounds, emphasizing down-weight, this is very different in the weimar video
C (timed=13-21)(timew=19-25)
<EW section traveling upstage, to the back
<RW Riley's movement is explicitly improvised, Brigel's is not. One of the only sections in the piece where my role had an explicit "this is improvisation" moment.
C (timed=22)(timew=26)
<EW is this different pose alignment?
<RW Not alignment, goes into counterpoint
U (timed=22-25)(timew= 27-31)
<EW seems that the previous alignment facilitates or starts this unison
C (timed=25-29)(timew32-36)
B (timed=28)
<EW brief pause in darmstadt video, seems like a still transition in the choreography, note how elasticized this timing has become in the weimar video
<RW Riley waits for Brigel to finish his movements and they then start again with a big exhale and back into our Counterpoint movement. Riley remembers when first learning the piece he would sometimes forget to wait for him and Brigel would have to remind him
C (timed=28 - 38)(timew=36-44)
AL (timed=38)(timew=44)
<EW shape alignment, end together in elbow pose
<RW Riley notes the alignment in the elbow position commences earlier, with a coordinated pre-movement
<EW this was not visible to Liz on the Darmstadt video until Riley showed her, on the weimar video it is more apparent
<RW the circular arm swiping movement that Liz didn't notice was modified by Riley-- the dancers didn't do it exactly how we they were taught but decided to do it individually differently, but with the same intentional memory of what it was. Brigel does it more similarly to how it "should be". Riley chose not to jump but to just use the arms
TT Brigel (timed=38-43)(timew=45-49)
<EW changes legs in elbow pose
<EW note, this is the so-called new beginning section

cue Riley (timed=43)(timew=49)
U (timed=43-53)(timew=49-59)
<EW movement named orange peel, this is one of the many repetitions of this motif, it is the first movement in the dance
cue Brigel (timed=44)
<RW This was an inherited verbal cue, meaning it was something the previous casts did that we adopted) (The explicitly verbal cues where Brigel says a name out loud (new beginning, surfer man joe, snake) are used for memory. The previous casts would loop and so one dancer would say to the other where they were in the piece to keep them on track. Riley almost always forgot to do "snake" (around 5:30) which means I rely on Brigel to tell me where I am.
B (timed=53)
<EW Riley takes one breath, seems like a rest
TT Riley (timed=53 - 59)(timew=59-65)
<EW Riley leg
cue Riley (timed=61)(timew=65)
<EW note riley turns his head, as if to send his sound back to his partner or to take peripheral peek, how do you approach this Riley?
<RW Riley takes as long as i want before inhale)
(Brigel is listening from his left ear primarily for Riley's long inhaled breath and then joins on the exhale)
U (timed= 61-72end)(timew=66-73)

Weimar Exposition
cue Riley (timew=10)
U (timew=10-14)
<EW this movement is named "shower head"
<RW inhale and exhale phase of movement, not looking each other, farthest away, too far away to actually hear each other, if we get that right, it starts the whole piece off right,
TT Riley (timew=14-20)
CS (timew=20)
<EW stomp sequence, riley makes a pre-stop, then the boys both stop, in unison or not
U (timew=20-22)
<RW run backwards to the space we have agreed to stop, depends on the space, not a set number of steps
B (timew=22-28)
<EW note the walks here are not required to be in unison
<RW not specified as to who decided to initiate the restart. in this case Brigel chose to go first
cue Riley (timew=28)
<RW here they can see and hear each other, much closer
U (timew=28-30)
<EW shower head
TT Riley (timew=31-57)
<EW i am confused about the stop sequence here
U (timew=56-58)
<EW run back
B (timew=59-65)
cue Riley **(**timew=66)
U (timew=67-68)
<EW shower head
TT Riley **(**timew=69)
U (timew=73-76)
<EW run back
B (timew=77-84)
<EW begin umpadump section
cue Riley (timew=85)
U (timew=86-99)
<EW Umpadump unison phrase
